# Enhancing Continuous Medication Safety Through e-Prescription and Clinical Decision Support Systems in Outpatient Practices and Pharmacies: Protocol for a Multiperspective Study (eRIKA Study)

**DOI:** 10.2196/87277

**Published:** 2026-04-07

**Authors:** Juliana Schmidt, David Lampe, Adriana Poppe, Ingo Meyer, Sara Söling, Juliane Köberlein-Neu, Daniel Grandt, Lara Düvel, Wolfgang Greiner, eRIKA Study Group, Franziska Folter

**Affiliations:** 1 Department of Health Economics and Health Care Management, School of Public Health, Bielefeld University, Universitaetsstrasse 25, Bielefeld, North Rhine-Westphalia, 33615, Germany; 2 PMV Research Group, Medical Faculty and University Hospital Cologne, University of Cologne, Cologne, North Rhine-Westphalia, Germany; 3 Centre for Health Economics and Health Services Research, Schumpeter School of Business and Economics, University of Wuppertal, Wuppertal, North Rhine-Westphalia, Germany; 4 Department of Internal Medicine, Klinikum Saarbrücken, Saarbrücken, Saarland, Germany; 5 Department Digital Care/Prevention, BARMER GEK, Wuppertal, North Rhine-Westphalia, Germany; 6 BARMER GEK, Wuppertal, North Rhine-Westphalia, Germany

**Keywords:** patient safety, medication therapy management, outpatient, clinical decision support system, polypharmacy, outcome and process evaluation, multiperspective, quasi-experimental design

## Abstract

**Background:**

Increased life expectancy is associated with increasing multimorbidity and polypharmacy, leading to a heightened risk of drug-drug interactions and adverse events, especially when multiple health care providers are involved. To address the urgent need for safer medication management in this population, tools such as medication plans (MP), electronic prescriptions (e-prescriptions), and clinical decision support systems (CDSS) offer valuable support. These instruments have the potential to enhance medication safety by providing physicians and pharmacists with a comprehensive overview of a patient’s overall medication regimen and by assisting health care professionals in making informed prescribing decisions.

**Objective:**

This study aims to improve medication therapy safety by combining e-prescriptions, the use of claims data, MPs, CDSS, and interprofessional communication. To comprehensively evaluate this complex intervention, a holistic multiphase study will be conducted, examining (1) the effectiveness of the intervention and (2) health-economic and (3) implementation-related aspects.

**Methods:**

A multiphase study design is used. In the first phase, the intervention is implemented in selected outpatient practices (n=10) and pharmacies (n=10) in 2 regions in Germany as part of a cluster-randomized controlled trial to assess process-related outcomes. The primary outcome is the congruence between the MP and claims data. In phase 2, the intervention is scaled up in 3 regions and evaluated in a quasi-experimental study. The required sample size for the intervention group is 3528 patients, with a synthetic control group matched from existing claims data. The primary outcome is a combined end point of all-cause mortality and hospitalization within 3 months of an index prescription. Quantitative methods (descriptive, regression-based methods using claims data, calculation of the incremental cost-effectiveness ratio, and survey-based analyses of implementation-related aspects) and qualitative methods (interviews and focus groups to capture experiences of health care professionals and patients) are used.

**Results:**

In phase 1, a total of 187 patients were recruited (74 in the intervention group and 113 in the control group) by June 2025. Phase 2 is currently ongoing, with data collection continuing through December 31, 2025. Final analyses are planned by March 2027.

**Conclusions:**

Medication safety in polypharmacy remains a critical challenge in Germany. This study provides multiperspective evidence supporting the nationwide implementation of the eRIKA (e-prescription as an element of interprofessional care pathways for continuous medication therapy management [*eRezept als Element interprofessioneller Versorgungspfade für kontinuierliche AMTS*]) intervention.

## Introduction

### Background

Recent demographic trends project a continued increase in the number of older adults [1]. This is closely linked to a growing global prevalence of chronic diseases [2], often accompanied by complex challenges, such as multimorbidity [3] and polypharmacy [4]. Polypharmacy, commonly defined as the concurrent use of 5 or more medications, is associated with various adverse outcomes [5,6], such as unwanted drug-drug interactions (DDIs), potentially leading to adverse drug events [7]. DDIs are even more likely if multiple providers are involved in prescribing medications to patients with polypharmacy [8]. For instance, a German study found that only 34.7% of patients treated with prescription medications receive their medications from a single physician [7], underscoring the challenges of managing chronic diseases.

A key challenge in managing polypharmacy is the health care providers’ lack of comprehensive knowledge of the patients’ current medications. This knowledge gap compromises both the safety and effectiveness of pharmacological treatment. To address this, the implementation of comprehensive medication plans (MPs) encompassing prescribed and over-the-counter (OTC) medications in a structured and accessible format for both patients and providers has been widely recommended [9,10]. Empirical evidence supports the positive impact of MPs on medication safety. For example, the use of MPs has been associated with reduced hospitalization rates and fewer incidents of medication misuse [11], as well as a slight decrease in negative DDIs [12].

Despite these benefits, the adoption and consistent use of MPs is still insufficient [7,13-15]. A major barrier is the frequent incompleteness of MPs. For example, a study conducted in Germany found that, in a regional sample, only 6.3% of the nationwide MPs met all formal requirements, including active substance, dosage, or reasoning [9]. Moreover, evaluations of the MP in Germany reveal discrepancies between documented and actual medication use in approximately 78% of cases, particularly regarding OTC medication and dietary supplements [16].

Another intervention promising improvement in medication therapy management is electronic prescriptions (e-prescriptions). They offer a way to significantly contribute to improvements, such as reducing the risk of medication errors and adverse events as well as improving efficiency in medication management if well designed [17,18]. The full potential of MPs and e-prescriptions can be realized when integrated into clinical decision support systems (CDSS). The benefits of CDSS in clinical practice have already been demonstrated in a number of disease conditions [19-22], and their application in medication safety is particularly promising. For example, CDSS can identify DDIs overlooked by physicians, thereby improving the quality of care [22]. Furthermore, CDSS may prompt physicians to engage in more critical and reflective prescribing practices [23,24].

### Objectives

The objective of the eRIKA study (e-prescription as an element of interprofessional care pathways for continuous medication therapy management [*eRezept als Element interprofessioneller Versorgungspfade für kontinuierliche AMTS*]) is to improve the safety of medication therapy through the combined use of e-prescriptions, claims data, MPs, CDSS, and interprofessional communication. To comprehensively evaluate the intervention, the study aims to investigate (1) its effectiveness as well as (2) health-economic and (3) implementation-related aspects.

## Methods

### Overview

This study protocol was written in accordance with the TIDieR (Template for Intervention Description and Replication) reporting checklist (Checklist 1) [25]. To comprehensively evaluate this complex intervention, a mixed perspective, multiphase study design will be employed. Phase 1 is designed as a cluster randomized controlled trial (c-RCT) with a relatively small sample size to allow for initial evaluation under controlled conditions. This is followed by a second phase involving a larger-scale quasi-experimental study with a matched control group (CG), scaling up the intervention and enabling the examination of the intervention’s effectiveness, cost-effectiveness, and implementation-related aspects in real-world settings.

### Intervention

The eRIKA intervention is designed to guarantee that for every patient receiving pharmacotherapy, an up-to-date and comprehensive electronic MP is generated, updated, and centrally stored, which can be accessed by patients, treating outpatient physicians, and pharmacists. Furthermore, medical information on the patient is electronically extracted from claims data provided by statutory health insurance (SHI) funds and is used to inform outpatient physicians about diagnoses, diagnostic procedures, and treatment history to date.

The eRIKA intervention is tested and implemented in participating outpatient practices and pharmacies. It is a digital software solution that integrates (1) the use of claims data in clinical practice, (2) the generation of an MP, (3) CDSS, (4) e-prescriptions, and (5) interprofessional communication.

Claims data from the SHI funds concerning prior prescriptions and selected clinically relevant information (such as diagnoses, medication prescription history, performed diagnostic and therapeutic procedures, treating outpatient physicians, hospital admissions and treatment, remedies, and aids) are fed into the software to ensure that MPs are complete and up to date at all times. This provides outpatient physicians with a comprehensive overview of the patient’s medical and medication history, which enables more efficient and safer medication prescriptions. The provision of medical and pharmaceutical information ensures that pharmacists have an overview of the patient’s current and past medications during their pharmaceutical consultations.

By using e-prescriptions via the eRIKA software and claims data, an MP is prefilled to be checked and manually supplemented with OTC medication, thus providing an up-to-date overview of the current medication regimens for use in outpatient practices and pharmacies. Furthermore, this MP is made accessible to patients, providing them with comprehensive information concerning their medication regimen.

Regarding CDSS, the eRIKA software also provides information and alerts for avoidable risks in medication therapy, for example, on contraindications, dosing errors, potentially inadequate medication for age, medication interactions, Dear-Doctor letters (*Rote-Hand-Briefe*), as well as guidance based on the guidelines for drug therapy in multimorbidity (*Leitlinie Arzneimitteltherapie bei Multimorbidität*), all of which are relevant to prescription decisions. Alerts are displayed in real time during prescription entry by the physician and are triggered automatically when rule-based checks detect a mismatch between the prescribed medication and patient-specific characteristics such as age, diagnoses, or concomitant medication. Alerts are categorized by severity (levels 1‐3) and can be addressed by modifying or canceling the prescription. Alternatively, physicians may override alerts if they consider the prescription clinically appropriate. For high-severity alerts (levels 2 and 3), physicians are required to actively acknowledge the alert and provide a brief justification, whereas lower-severity alerts may be acknowledged without justification. All alerts, physician responses, and overrides are automatically documented within the system, enabling assessment of alert exposure and handling during the intervention. A tailored subset of alerts relevant for pharmaceutical counseling is presented to pharmacists.

e-Prescriptions via the eRIKA software are issued in accordance with the legal requirements under German law.

Regarding interprofessional communication, the software further enables the outpatient physician to document remarks regarding the selected medication and assign corresponding levels of urgency.

**Figure 1.**
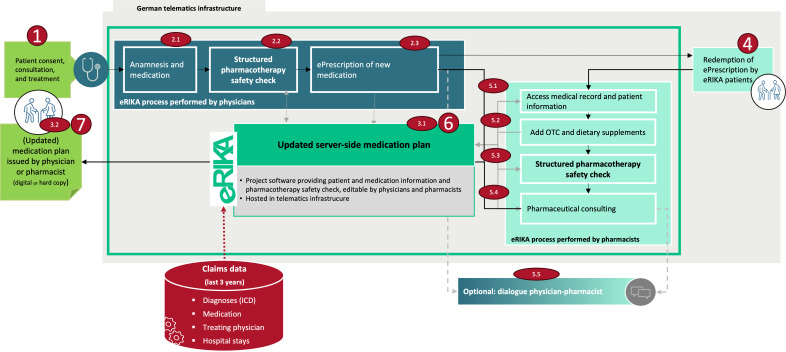
Functionalities and processes of the eRIKA (e-prescription as an element of interprofessional care pathways for continuous medication therapy management [*eRezept als Element interprofessioneller Versorgungspfade für kontinuierliche AMTS*]) intervention including the German telematics infrastructure, physician-led medication review, server-sided updated medication plans, and pharmacist-led medication review. ICD: International Statistical Classification of Diseases and Related Health Problems; OTC: over-the-counter.

An overview of the structured eRIKA process is provided in Figure 1. Following patient consent (1), outpatient physicians use the eRIKA software during routine consultations to access claims-based medical information and the centrally stored MP. After conducting the anamnesis and reviewing the current medication (2.1), supported by CDSS alerts (2.2), new medications are prescribed electronically (2.3). Issued e-prescriptions and imported claims data automatically update the server-side MP (3.1). Furthermore, an up-to-date MP is provided to the patient as a printout (3.2). Next, patients redeem their e-prescriptions in participating pharmacies (4). In this step, pharmacists access the MP and relevant patient information (5.1) and add OTC medications and dietary supplements to the MP if necessary (5.2). The pharmacist-led structured pharmacotherapy safety check is supported by a tailored set of alerts relevant to pharmaceutical counseling (5.3). Pharmacists provide medication counseling (5.4) and, if necessary, contact the treating physician (electronically or via telephone) regarding medication-related remarks or substitution proposals for review (5.5). Following optional physician review, the MP is updated accordingly and made available to all involved health care professionals via the eRIKA software as the server is updated automatically (6), as well as to patients in printed form (7).

Reimbursement is tied to the completion of defined process steps to support adherence to the intended process. Physicians had to fulfill the following five steps: patient enrollment (1); conducting the anamnesis (2.1) and a structured safety check (2.2) and issuing e-prescriptions (2.3); providing an updated MP (3.1 and 3.2); pharmacists dispensing at least 1 e-prescription (4); and reviewing the corresponding risk analysis (5.1), documenting any required modifications to the MP (5.3), and issuing an updated MP if necessary (7).

Physicians and pharmacists received initial training sessions during which the software developer explained the system’s functionality, providing opportunities to ask questions and report technical issues. In addition, a brief process manual was integrated directly into the software for ongoing reference.

### Phase 1: Cluster Randomized Controlled Trial

#### Study Design

Process-related outputs and outcomes are evaluated by a c-RCT from October 2023 to June 2024 following the corresponding extended CONSORT (Consolidated Standards of Reporting Trials) guidelines [26]. Randomization is performed at the cluster level, with each cluster comprising an outpatient practice and at least one geographically proximate pharmacy, assigned to either the intervention group (IG) or the CG. Patients in clusters assigned to the IG are treated with the eRIKA intervention, while patients in the CG receive treatment as usual. Due to the nature of the intervention, blinding is not feasible; thus, health care providers, patients, and outcome analysts are all aware of group allocation.

#### Eligibility Criteria

Patients (1) aged 18 years or older, (2) insured by a participating SHI fund (BARMER, AOK Nordost), and (3) taking 3 or more long-term prescription medications are included in the study. This includes patients receiving their third medication prescription during the outpatient consultation leading to study participation, thus fulfilling the third criterion from that point onward. Patients participating in the eLiSa (electronic Life Saver) project are excluded from the study. No further specific exclusion criteria are applied.

Outpatient practices are included. No further inclusion or exclusion criteria on the outpatient practice and pharmacy level are applied.

#### Study Setting and Recruitment

The eRIKA intervention is implemented in selected outpatient practices and pharmacies in 2 regions (Berlin and Westphalia-Lippe). The outpatient practices are selected and approached via the Associations of Statutory Health Insurance Physicians Westphalia-Lippe and the Association of Berlin Physician Networks (*Arbeitsgemeinschaft Berliner Arztnetze*) for the Berlin area. Pharmacies are recruited via the SHI funds in cooperation with regional pharmacist associations. Patients are recruited by their treating outpatient physician.

#### Outcomes and Measures

##### Primary Outcome

The primary outcome of phase 1 is the congruence of the MP and claims data (OTC not considered), which is operationalized as the ratio of the medications listed in the MP and in claims data (numerator) to all documented medications according to claims data or MP (denominator). Thus, values range between 0 and 1, with a higher value being associated with a greater alignment between the MP and the medications documented in claims data.

##### Additional Evaluations

The primary end point is complemented by a process evaluation and a socioeconomic impact assessment (SEIA), each playing a key role in the sustainable adaptation and implementation of the intervention in phase 2 and beyond the study (Table 1).

**Table 1.** Overview of additional formative and socioeconomic evaluation of the eRIKA (e-prescription as an element of interprofessional care pathways for continuous medication therapy management [*eRezept als Element interprofessioneller Versorgungspfade für kontinuierliche AMTS*]) intervention in phase 1, including outcomes, data sources, and assessment time points.

Outcome	Data source	Time point
Formative evaluation		
Acceptability, appropriateness, and feasibility [27]	Quantitative data from surveys	Baseline and after 6 months
Adoptability, implementability, and sustainability	Quantitative data from surveys	Baseline and after 6 months
Context characteristics	Quantitative data from surveys	Baseline and after 6 months
Barriers and facilitating factors	Qualitative data from interviews	End of the c-RCT^a^
Intervention fidelity	User data of the software	Continuously over the study period
Socioeconomic impact assessment		
The time needed for implementation correlates positively with expected benefits	Literature research, MP^b^, claims data, primary data, and software data	Patient-specific

a c-RCT: cluster randomized controlled trial.

b MP: medication plan.

### Data Collection

#### Primary Data

Once the patient has provided informed consent, the MP is transmitted to a trust center. In the IG, the software provider extracts the MPs from the software solution and transmits them electronically to the trust center, whereas in the CG, the treating outpatient physician sends the MP via postal mail. The trust center digitizes the MP (for the CG) and subsequently links it with the software and claims data.

Outcomes related to the formative evaluation are obtained from participating outpatient physicians and pharmacists from both study groups through 2 surveys (baseline and after 6 months) and qualitative interviews.

#### Claims Data

Claims data from September 2023 to December 2024 are used in the analysis. This includes data for the duration of the c-RCT, as well as a preobservation period of 1 year to establish a baseline for the primary end point. The required claims data from participating SHI funds are specified in a coordinated minimal dataset.

#### Software Data

For the data derived from the software solution used to evaluate the primary end point, the SEIA, and user behavior and interaction with the software (formative evaluation), a minimal data set is coordinated with the software provider.

#### Data Linkage

Data are linked by the trust center. Claims data and software data are linked with the patients’ MP data based on a pseudonymized study ID.

### Sample Size

For the evaluation of the primary outcome, 10 clusters of outpatient practices and pharmacies in geographical proximity are randomized into 2 groups (5 IG and 5 CG). Because, to our knowledge, no comparable studies are available to support plausible assumptions regarding the primary outcome, a moderate Cohen *d* effect size of 0.5 is assumed, in line with established conventions for studies with novel outcomes [28,29]. The assumed intracluster correlation coefficient (ICC) of 0.05 is based on the evidence from c-RCTs conducted in similar primary care and outpatient settings, where ICCs typically range from 0.01 to 0.10 [30-32]. Given these assumptions, an α-error of .05 (2-sided), and a power of 0.8, the overall sample size is 138 (2×69). Based on experience from prior studies in comparable outpatient and primary care settings [33,34], as well as to account for possible patient withdrawal and loss of analyzable primary outcome data due to incomplete or manually transmitted MPs, a dropout rate of 40% is expected, increasing the sample size to 230 patients (2×115). The resulting number of patients per practice is 23.

### Analyses

#### Primary Outcome

The evaluation of the primary outcome is initially descriptive. In the next step, appropriate inferential statistical methods (eg, 2-tailed *t* tests, linear mixed models [LMMs], generalized LMMs [GLMMs], or generalized estimating equations to control for potential confounding factors, cluster effects via random intercepts, and, if applicable, repeated measures within individuals) are applied [35]. Analysis is adjusted for multiple testing using the Bonferroni-Holm correction.

#### Process Evaluation

All process evaluation data are initially analyzed descriptively and exploratively. Qualitative data material is analyzed by combining a broadly deductive analytical approach with an inductive analysis to explore new findings related to the specific intervention and context.

#### Socioeconomic Impact Assessment

**Figure 2.**
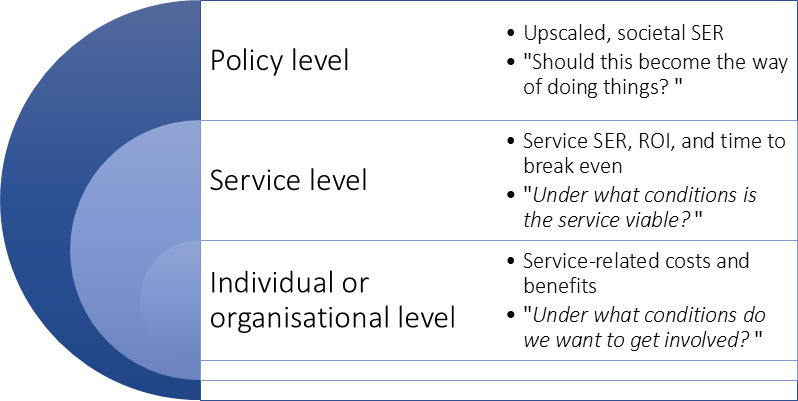
Levels of the socioeconomic impact assessment (SEIA), illustrating policy, service, and individual or organisational perspectives, along with corresponding evaluation questions. ROI: return on investment; SER: socioeconomic return.

Sustainability planning for the integration of the intervention into standard care is informed by a SEIA. It is a formative evaluation that aims to answer questions on 3 levels that are visualized in Figure 2.

The SEIA methodology is based on cost-benefit analysis as defined by Drummond et al [36] and the recommendations of the UK HM Treasury [37], the Federal Government Commissioner for Information Technology [38], and the White House Office for Management and Budget [39]. An already established framework and associated evaluation software, developed for business model development for IT-based utility services, is applied [40].

### Phase 2: Quasi-Experimental Study

#### Study Design

A quasi-experimental study with an intervention or observational period of 15 months is conducted from July 2024 to December 2025. Eligible IG patients are recruited in the field and treated with the eRIKA intervention, while the CG receives treatment as usual and is derived from claims data using appropriate matching methods. Neither health care providers, patients, nor analysts are blinded to treatment allocation.

#### Eligibility Criteria

The study population in phase 2 is selected according to the eligibility criteria applied in phase 1. In addition, phase 2 includes a separate study arm comprising patients who take 5 or more long-term prescription medications.

#### Study Setting and Recruitment

The eRIKA intervention is implemented in participating outpatient practices and pharmacies in 3 regions (Berlin, Saarland, and Westphalia-Lippe). Recruitment of IG outpatient practices and pharmacies is carried out by the collaborating regional Associations of Statutory Health Insurance Physicians, the Association of Berlin Physician Networks (*Arbeitsgemeinschaft Berliner Arztnetze*), and regional pharmacist associations. The University of Wuppertal contacts participating outpatient practices and pharmacies for surveys and qualitative interviews. Outpatient practices and pharmacies involved in phase 1 (IG and CG) are invited to participate in the IG in phase 2. IG patients are recruited by their treating outpatient physician. Recruitment monitoring is implemented in both study phases to enable early identification of deviations and the timely initiation of supportive recruitment measures. The IG is assigned a data-derived CG through the application of appropriate matching methods.

#### Outcomes and Measures

##### Primary Outcome

The primary outcome of phase 2 is a combined end point of the incidence of all-cause mortality or all-cause hospitalization in patients with polypharmacy 3 months after an index prescription at baseline.

##### Additional Evaluations

With the secondary outcomes in phase 2, the eRIKA intervention is evaluated in terms of a summative, socioeconomic impact and health economic evaluation, partly building on the outcomes from phase 1 (SEIA). A comprehensive overview of the secondary outcomes and aspects of the process evaluation in phase 2 is shown in Table 2.

**Table 2.** Overview of additional processes, secondary summative outcomes, and socioeconomic evaluations of eRIKA (e-prescription as an element of interprofessional care pathways for continuous medication therapy management [*eRezept als Element interprofessioneller Versorgungspfade für kontinuierliche AMTS*]) in phase 2, including outcomes, data sources, and assessment time points.

Outcome	Data source	Time point or period
Process evaluation		
Acceptability, appropriateness, and feasibility [27]	Quantitative data from surveys	Provider-specific: at baseline, after 3 months and up to 12 months after initial implementation
Adoption, penetration, and sustainability	Quantitative data from surveys	Provider-specific: at baseline, after 3 months and up to 12 months after initial implementation
Context characteristics	Quantitative data from surveys	Provider-specific: at baseline, after 3 months and up to 12 months after initial implementation
Interprofessional collaboration	Qualitative data from interviews and focus groups	Provider-specific: After six months and at the end of phase 2
Intervention fidelity	User data of the software	Continuously over the study period
Secondary summative outcomes		
Congruence of the MP^a^ and claims data	Claims data	Patient-specific: at baseline, follow-up quarter
Adverse drug event-related diagnoses and health care resource use (inpatient or outpatient)	Claims data and software data	Patient-specific: continuously over the study period
Potentially inadequate medications	Claims data and software data	Patient-specific: continuously over the study period
Socioeconomic impact assessment		
Socioeconomic return	Literature research, MP, claims data, primary data, and software data	Provider-specific: at baseline, after 3 months and patient-specific: observation period of 3 months
Health economic evaluation		
Health care resource use	Claims data	Patient-specific: observation period of 3 months
Health care costs	Claims data	Patient-specific: observation period of 3 months

a MP: medication plan.

### Data Collection

#### Primary Data

Prior to the intervention, the treating outpatient physician transmits the MP to the trust center via postal mail. As part of the patient-specific follow-up assessment in the subsequent quarter, the software provider will extract the MP and transmit it electronically. The linkage and digitization of the MP are carried out in the trust center. MPs are not available for the matched CG.

The outcomes related to the process evaluation are collected from the participating outpatient physicians and pharmacists using a longitudinal survey (3 time points; Table 2), qualitative interviews (at least 10 professionals per region), and focus groups (2 focus groups with 8‐10 professionals and interviews with at least 15 patients).

#### Claims Data

Although the intervention runs from July 2024 to December 2025, claims data from January 2023 to December 2025 is used in the analysis, covering the per patient–specific preintervention period as well as the intervention period, including the last patient follow-up of 3 months. In addition to the patient-specific observation period, this includes a preinterventional phase to adjust for preinterventional disease burden and costs. The required claims data from participating SHI funds are specified in a jointly defined minimal dataset.

#### Software Data

For selected secondary end points, such as the congruence between claims data and MP, as well as user behavior and interaction with the software (process evaluation), a minimal dataset is defined in collaboration with the software provider.

#### Data Linkage

Data linkage is performed in the same manner as in phase 1.

### Sample Size

For the outcome evaluation, the incidence of all-cause hospitalization or all-cause mortality (primary outcome) for patients with polypharmacy is estimated to be 32.9%, based on a preliminary analysis of BARMER claims data. Given the inconsistent evidence regarding the impact of medication management interventions on outcomes such as hospitalization and mortality [11,33,41-45] and the fact that these outcomes are influenced by multiple clinical and contextual factors beyond the intervention itself, a conservative absolute risk reduction of 4 percentage points was assumed, corresponding to a reduction to 28.9%. The evaluation is carried out for 2 groups, each in comparison to a matched CG: for patients with (1) 3 to 4 medications and (2) 5 or more medications. With an α-error of .05 and a power of 1-β=0.80, based on a 2-sided Fisher exact test (for binary outcomes), this yields a required sample size of 1411 patients per group, resulting in a total of 2822 patients. Accounting for a dropout rate of 20%, the required sample size is adjusted to 3528 participants.

Drawing on experience from previous projects, an average of 10 to 20 patients per practice is expected, corresponding to approximately 175 to 350 outpatient physicians and 116 to 233 outpatient practices and corresponding pharmacies.

The IG is assigned a data-derived CG by selecting and matching comparable insured individuals from the claims data provided by the participating SHI funds. These individuals meet the study’s inclusion criteria and have not received treatment in any of the participating outpatient practices or pharmacies during the intervention phase. An iterative matching approach is employed, consisting of a primary procedure and alternative procedures. If the primary method of direct matching based on relevant matching criteria (eg, age, gender, and prescribed medications) does not yield a sufficiently large and balanced matched CG, an alternative procedure based on propensity score matching is applied, using relevant matching criteria as covariates. This approach aligns with standard practice in the application of matching techniques [46], as there is no single optimal choice of matching approach for any given analysis [47]. To assess residual confounding after matching, covariate balance between the IG and the matched CG is evaluated using standardized mean differences and visual diagnostics for all matching variables and key preintervention characteristics (eg, health care use and comorbidity indicators) [48,49]. To address any residual imbalance, outcome analyses are conducted using regression models adjusted for baseline covariates that remain imbalanced after matching. As a sensitivity analysis, alternative weighting approaches are applied to assess the robustness of the results to the choice of matching method [50].

### Analyses

#### Summative Evaluation

The evaluation uses claims data encompassing patients in the IG, as well as a synthetic CG. Primary and secondary end points are initially analyzed descriptively. Subsequently, inferential statistical analyses are conducted using LMMs, GLMMs, or generalized estimating equations, accounting for independent covariates such as age, gender, and comorbidity, as well as for repeated measurements within individuals and cluster effects via random intercepts [35]. Results are adjusted for multiple testing using the Bonferroni-Holm correction.

#### Health Economic Evaluation

Using claims data, the health economic evaluation is conducted on a per-patient basis from a third-party payer perspective (SHI in Germany), excluding societal costs such as productivity losses. The analysis encompasses health care use and costs related to outpatient, inpatient, rehabilitative and nursing or home care, pharmaceuticals, therapeutic devices, nonphysician specialist services, as well as patient transportation services and SHI-financed sick leave or pay. Intervention-related costs are also included. To quantify the impact of the intervention on total costs, appropriate descriptive methods (eg, mean, median, and SD) are used. As cost data are typically right-skewed and may include extreme values, appropriate regression-based methods such as LMM or GLMM with suitable distributional assumptions (eg, gamma with log-link or log-normal distribution) are applied. Extreme cost values are initially retained and checked for implausibility, and the influence of extreme observations is examined. Relevant variables (eg, prior-year costs) are included to control for potential confounding. To account for clustering of individuals within outpatient practices or pharmacies, cluster-level random intercepts will be incorporated into the model. Cost-effectiveness is determined by means of the incremental cost-effectiveness ratio, calculated by dividing the difference in costs by the difference in health benefit in the IG compared to CG. The effectiveness of the intervention is measured by the primary outcome (hospitalization and/or death). Appropriate sensitivity analyses (eg, deterministic or probabilistic) [51-54] are conducted to account for uncertainties and evaluate their impact on the results.

#### Process Evaluation

Initially, data are analyzed descriptively and exploratorily. For the qualitative data material, either a content analysis or a qualitative-descriptive analysis is used. A 2-stage procedure is applied. First, deductively, interview paraphrases are assigned to the predefined themes and subthemes of the underlying frameworks or (implementation) theories (eg, innovation readiness, consolidated framework for implementation research, and normalization process theory). Building on this, an inductive processing of the content will occur within these themes. Questionnaire and software data are analyzed descriptively and exploratorily.

#### Socioeconomic Impact Assessment

The sustainability planning for the transition from phase 1 to standard care continues in phase 2. This process follows the methodology outlined earlier (see *Socioeconomic Impact Assessment* under *Phase 1: Cluster Randomized Controlled Trial* section).

### Ethical Considerations

For phase 1, ethical approval was granted by the ethics committee of the Medical Association of Westphalia-Lippe (2023‐526-f-S). For phase 2, ethical approval was granted by the ethics committee of the Medical Association of Westphalia-Lippe (2024‐599-f-S) and the Medical Association of Saarland (208/24).

This study is conducted in accordance with the Declaration of Helsinki. The CG in phase 2 is derived from secondary pseudonymized claims data. Informed consent from CG individuals is not obtained. However, the study poses minimal risk, and a waiver of informed consent for the use of pseudonymized secondary data was reviewed and approved by the responsible ethics committee as well as regulatory authorities. All data are collected, pseudonymized, and stored in accordance with German General Data Protection Regulation legislation based on the European General Data Protection Regulation.

Patients in phase 1 and IG patients in phase 2 voluntarily provide written informed consent prior to participation. Participants are informed about the project, the evaluation procedures (including data linkage of MP, software data, and claims data), participation requirements, and their rights. In phase 2, patients additionally consent to participate in interviews. By signing a separate consent form, they agree to be contacted by research staff. No financial compensation is provided to patients in the study.

Participating outpatient practices and pharmacies consent to participate through their project participation agreement, replacing the need for individual staff consent forms. Study staff are informed about study procedures prior to each data collection activity. By completing questionnaires or voluntarily participating in interviews or focus groups, staff consent to the project-related processing, use, and storage of their data.

All participants may withdraw their informed consent at any time without providing reasons and without facing any disadvantages. In the event of withdrawal, no further data will be collected, and previously collected data will be anonymized in accordance with data protection regulations.

Study data are stored in encrypted form with access restricted to authorized project staff. Personal contact data are stored separately from research data and deleted in accordance with data protection regulations.

The protocol was registered with the German Clinical Trials Register database (phase 1: DRKS00032777 and phase 2: DRKS00035030).

## Results

**Figure 3.**
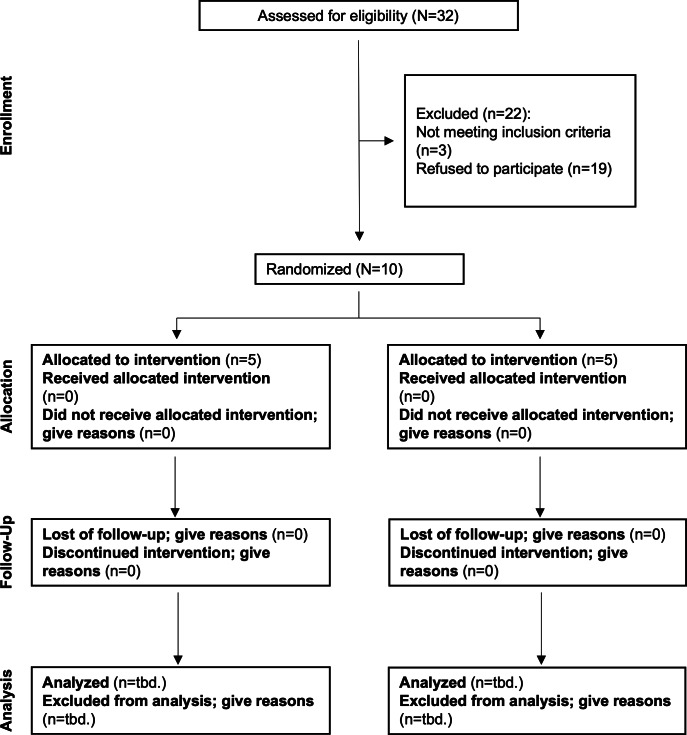
Flow diagram of the progress of clusters and individuals through the phases of the cluster randomized eRIKA (e-prescription as an element of interprofessional care pathways for continuous medication therapy management [*eRezept als Element interprofessioneller Versorgungspfade für kontinuierliche AMTS*] study (September 2025). tbd.: to be determined.

The study is currently in phase 2. The data collection for phase 1 was completed by June 30, 2025. In total, 187 patients were successfully recruited for phase 1, comprising 74 individuals in the IG and 113 in the CG (CONSORT flowchart of the progress of clusters and individuals through the phases of a c-RCT in Figure 3). As of October 2025, 344 participants have been enrolled for phase 2. Data collection for this phase will continue through December 31, 2025. Due to a time lag in claims data provision, analyses for both phases will be completed by March 2027.

## Discussion

### Principal Findings

There is an urgent need to improve the safety of medication therapy for patients with polypharmacy. The growing number of older adults is accompanied by rising multimorbidity and polypharmacy [1-4], increasing the risk of DDIs [5-7], particularly when multiple providers are involved [8]. Meanwhile, MPs often remain incomplete or underutilized [7,13-15]. Structured MPs and CDSS have shown positive effects on medication therapy safety, such as reducing DDIs, hospitalizations, and inappropriate medication use [11,12]. However, the widespread implementation of MPs remains limited, highlighting the urgent need for better integration into routine care. The eRIKA software is a promising intervention to improve medication therapy safety for patients with polypharmacy. The combined use of e-prescriptions, claims data, automatically generated MPs, and CDSS has the potential to provide health care providers with an up-to-date overview of the current medication, contribute to the completeness and actuality of the available MP, and support outpatient physicians and pharmacists in their treatment or consultation process. The eRIKA intervention extends forms of care that have already been tested and evaluated in other projects such as the AdAM (Anwendung für digital unterstütztes Arzneimitteltherapie-Management) [33] or TOP (Transsektorale Optimierung der Patientensicherheit) [34] project by adding the use of e-prescriptions and emphasizing the essential role of pharmacists.

### Strengths

A key strength of the study is the alignment of the eRIKA project with the Medical Research Council (MRC) framework for the development and evaluation of complex interventions [55]. This includes a comprehensive approach encompassing intervention development, implementation, and multilevel evaluation, including formative components. Furthermore, the study is structured in 2 phases to ensure both rigor and scalability. Phase 1 is conducted as a c-RCT, allowing for a controlled assessment of the intervention’s feasibility within a clinical setting. In phase 2, the study involves a larger sample size to evaluate the effectiveness and large-scale implementation. Moreover, with this comprehensive analysis approach, a multitude of relevant outcomes for the effectiveness of the eRIKA software and process are addressed, as recommended by the literature [56]. The intervention itself is intersectoral in nature, involving coordinated efforts across different sectors of the health care system, which enhances its practical relevance.

### Limitations

The study faces limitations that may affect the interpretation and implementation of its findings. In phase 1, the small number of clusters may reduce the generalizability of the results. In addition, the primary outcome in phase 1 (congruence between MP and claims data) is novel, and therefore the underlying effect size and ICC assumptions are based on methodological conventions rather than outcome-specific empirical estimates. Furthermore, claims data are subject to delays and omissions and may not fully reflect actual medication use. Observed discrepancies between the MP and claims data may therefore result from timing differences rather than true inconsistencies, which should be considered when interpreting the congruence outcome in phase 1. Phase 2 is designed as a quasi-experimental study, which, while useful for evaluating interventions in more naturalistic settings, is not exempt from potential confounding and bias. Rigorous (matching) methods are necessary to mitigate such risks. Additionally, the implementation of the intersectoral interventions may present practical challenges, including reliance on service providers, variations in stakeholder engagement, coordination between sectors, technical challenges (at the point of installation or software usage), and contextual factors that could influence intervention fidelity and outcomes. Particularly the implementation of other digital interventions on a national level, such as e-prescriptions in general or the national rollout of electronic patient records, might hinder physicians’ recruitment efforts and intervention fidelity. These factors will be considered in the formative evaluation as well as in the statistical analysis by using a time variable.

### Conclusions

Improving medication safety for patients with polypharmacy remains a critical health care priority in Germany. This study provides a comprehensive, multiperspective evaluation of the eRIKA intervention, delivering robust evidence to support its nationwide implementation.

## Supplementary material

10.2196/87277 Checklist 1 TIDieR checklist.

## Abbreviations

c-RCT: cluster randomized controlled trial
CDSS: clinical decision support system
CG: control group
CONSORT: Consolidated Standards of Reporting Trials
DDI: drug-drug interaction
e-prescription: electronic prescription
eLiSa: electronic Life Saver
eRIKA: e-prescription as an element of interprofessional care pathways for continuous medication therapy management (*eRezept als Element interprofessioneller Versorgungspfade für kontinuierliche AMTS*)
GLMM: generalized linear mixed model
ICC: intracluster correlation coefficient
IG: intervention group
LMM: linear mixed model
MP: medication plan
MRC: Medical Research Council
OTC: over-the-counter
SEIA: socioeconomic impact assessment
SHI: statutory health insurance
TIDieR: Template for Intervention Description and Replication
